# Beyond the Halo: Molecular Mechanisms and Ecological Aspects of the Infection Strategy of *Pseudomonas savastanoi* pv. *phaseolicola*

**DOI:** 10.3390/ijms27156729

**Published:** 2026-07-28

**Authors:** Mateusz Wala

**Affiliations:** Department of Plant Physiology and Biochemistry, Faculty of Biology and Environmental Protection, University of Lodz, Banacha 12/16, 90-237 Lodz, Poland; mateusz.wala@biol.uni.lodz.pl

**Keywords:** halo blight, phaseolotoxin, *Phaseolus vulgaris*, plant–microbe interactions, *Pseudomonas savastanoi* pv. *phaseolicola*

## Abstract

*Pseudomonas savastanoi* pv. *phaseolicola*, the causal agent of halo blight of bean, is a useful model for studying bacterial adaptation to plant infection. Although its toxins, effectors, population dynamics, and epidemiology have been investigated for decades, these aspects have often been treated separately. This narrative synthesis reinterprets the available literature within a stage-based infection framework encompassing host-surface colonization, establishment of a compatible apoplastic habitat, evasion of host immune responses, multiplication, and spread to another compatible host. Particular attention was given to studies published after the last major review on this pathovar, whereas older studies were retained when they represented primary reports, classical experimental evidence, or information that has not been superseded. The review shows that successful infection is best understood as involving opportunistic entry, rapid transition from an epiphytic to a pathogenic lifestyle, effector-dependent immune suppression, phaseolotoxin-mediated manipulation of host primary metabolism, the exploitation or establishment of hydrated apoplastic conditions, and environmentally driven dissemination. It also distinguishes experimentally supported mechanisms from unresolved or hypothesis-generating areas, including stomatal reopening, micronutrient acquisition, population-level division of labor, alternative hosts, and symptomless reservoirs. The major synthesis emerging from this review is that the infection biology of *P. savastanoi* pv. *phaseolicola* cannot be reduced to individual virulence factors because disease development depends on how these factors are coordinated with host-derived resources, immune constraints, and environmental opportunities.

## 1. *Pseudomonas savastanoi* pv. *phaseolicola* and Halo Blight

The etiological agent of halo blight (*HB*) of bean (*Phaseolus vulgaris* L.) was described a century ago [1]. Since then, its taxonomical classification has changed multiple times. It was classified as a *Pseudomonas syringae* pathovar (pv.) *phaseolicola* for a long time [2], but in accordance with the last taxonomical revision of the genus, the etiological agent of *HB* is now treated as *Pseudomonas savastanoi* pv. *phaseolicola* (*Psp*), with no change in the biological circumscription, host association, or diagnostic concept of the taxon [3], allowing debate on this species without additional considerations of its taxon concept. Taxonomic relationships within the *P. syringae* species complex remain entangled and have been repeatedly reassessed [4,5,6]. To date, precise taxonomic placement remains debated.

Many approaches are currently used for the identification, diagnosis, and characterization of new *Psp* isolates [7,8]. Notably, most molecular assays applied in this context are primarily tools for detection, identification, or strain-level characterization rather than de novo taxonomic resolution. Although molecular methods have become central to accurate diagnosis, some phenotypic tests remain valuable [2,9]. For example, the biochemical/nutritional LOPAT test [9] supports the preliminary identification of likely *Psp* isolates by demonstrating levan production on sucrose medium, the absence of oxidase activity, the absence of arginine dihydrolase activity, and the absence of pectolytic activity on potato slices or pectate gel, together with a tobacco hypersensitivity response [2]. Other classical phytopathological tests are useful for studying intraspecific phenotypic diversity. In particular, most *Psp* isolates can be assigned to nine races, designated 1–9, on the basis of the hypersensitive response and disease symptom development in eight common bean cultivars [2,10]. However, because some isolates cannot be assigned to any of the proposed races, molecular assays are often used to support more accurate diagnosis and identification of *Psp* [11], and they are now widely applied in this context [7,8]. Nevertheless, race-based systems remain widely used, especially in studies of host resistance and disease epidemiology [12,13,14].

Despite decades of research, *HB* remains among the most important bacterial diseases of beans. More broadly, diseases caused by phytopathogenic pseudomonads are among the most economically important bacterial diseases of plants [15], which highlights the need for further studies.

## 2. Plant Infection and Attack Strategy

For the purpose of this review, successful infection is operationally resolved into five partially overlapping functional challenges: (1) colonizing host tissues, (2) finding or establishing a compatible habitat inside the host organism, (3) evading its immune response, (4) multiplying, and (5) spreading to another compatible host. This general structure follows broad infection biology logic [16], and it is used here as an analytical framework rather than a specialized disease-cycle linear model. The reason for this choice is that the review focuses on the molecular, physiological, and ecological traits of *Psp* according to the functions they perform during infection rather than on the descriptive sequence of inoculum production, penetration, symptom development, survival, and epidemiological recurrence. Each of these steps, which are central to this review, can be limited by plant immune responses, physical barriers, or environmental constraints. Therefore, successful infection by this pathogen cannot be reduced solely to overcoming host defense mechanisms but involves the coordinated resolution of several overlapping biological challenges. Individual processes may contribute to more than one stage, and some stages may occur simultaneously or only in particular bacterial subpopulations. Rather than organizing current knowledge by virulence factor class, this review adopts the abovementioned framework to determine which traits of *Psp* contribute to each stage of infection. Since the last review on this topic [2], studies on *Psp* have continued to address effector-triggered outcomes and race evolution while also increasingly considering coordinated habitat construction and population-level behavioral heterogeneity. Thus, a central question of this review is how *Psp* integrates its own molecular repertoire with host-derived molecules and environmental opportunities to complete the infection cycle. Additionally, this review is intentionally pathogen-centered; host genetic determinants are addressed only where strictly necessary to interpret the strategy of the pathogen or outline promising research directions.

This article is a narrative and synthetic review rather than a systematic review. The literature was selected and interpreted according to its relevance to the stage-based infection framework used here. Particular attention was given to studies published after the last major review on this pathovar [2], whereas older studies were retained when they represented primary reports, classical experimental evidence, or information that has not been superseded. The synthesis focuses on studies addressing halo blight, *P. savastanoi* pv. *phaseolicola* (including synonymous names), toxins, effectors, nutrition and physiology of this pathogen, population dynamics, environmental regulation of disease, alternative hosts, and disease dissemination. Findings from related phytopathogenic *Pseudomonas* pathovars were considered only when direct evidence for *Psp* was limited and when they helped to define plausible mechanisms or unresolved research questions.

## 3. Colonization of Host Tissues

The colonization of host tissues is limited by physical preinfective mechanisms [17]. The primary preinfective barrier against invaders (cell covers, i.e., the cuticle and upper cell wall of the epidermis) is efficient only as long as it is not subjected to mechanical disruption (caused by fungal pathogens or abiotic stresses) or enzymatic degradation (caused by both fungal and bacterial pathogens [17]). It has been shown that the genome of *Psp* contains open reading frames (ORFs) for two cellulases, two pectate lyases, one pectin lyase, and one polygalacturonase [18]. All these enzymes can be secreted by the type two secretion system (T2SS [18]). Some studies have indicated that the induced activity of pectolytic enzymes in plant tissues is typical during the development of *HB* [19]. Many other lines of evidence suggest that degradation or remodeling of the cell wall is an important component of the pathogenic lifestyle of *Psp*. Other phytopathogenic pseudomonads are able to produce only a limited number of these enzymes; for example, *P. syringae* pv. *lachrymans* produces only one pectate lyase [20]. More importantly, in vitro transcriptomic studies revealed that the transcription of pectin lyase (*pnIA*) and polygalacturonase (PSPPH_A0072) was threefold and twofold greater, respectively, when *Psp* cultures were treated with common bean tissue extracts [21]. Together, these observations indicate that *Psp* possesses the genetic capacity for cell wall degradation or remodeling and that some genes encoding cell wall-degrading enzymes are transcriptionally responsive to host-derived cues. However, direct loss-of-function evidence demonstrating that individual *Psp* cell wall-degrading enzymes contribute to virulence in planta is currently lacking. Therefore, the role of these enzymes in *HB* should be treated as a plausible component of the infection process and requires further validation.

However, constitutive forcing of passive barriers just by enzymatic digestion from the surface of colonized organs as an initial phase of infection would be inefficient because of potentially high costs for such an attack strategy. Therefore, phytopathogenic pseudomonads, including *Psp*, enter host tissues through stomata or wounds [2]. This event seems to be strongly regulated by environmental conditions, including rainfall quality and intensity and total volume of precipitation, causing the opening of stomata or perturbations in plant protective barriers [22]. Additionally, the chemical composition of rainfall influences the first stage of the *HB*; acidic rainfall can affect the colonization stage because of direct pH-based influences on bacterial epiphytic populations and the extensive erosion of cuticular waxes [23]. More often, severe rainfall strongly favors the epiphytic-to-pathogenic transition of *Psp* and is consistent with increased entry through natural openings, notably stomata [22]. These findings are in line with observations of other pathogens showing massive aggregation of bacteria at stomata during the first steps of infection [24,25]. In *Psp*, this is governed by pili; piliated bacteria are able to attach to the leaf surface at the site of the stomata, whereas nonpiliated bacteria are evenly distributed on the leaf surface [26].

The ability of pathogenic bacteria to enter plant tissues through stomata is usually highly limited because stomatal defense strongly constrains entry due to the fast and efficient recognition of invaders based on microbe-associated molecular patterns (MAMPs) and the ability to employ pattern (MAMP)-triggered immunity (PTI [27]). The common event triggering PTI during bacterial disease, which is likely important for motile *Psp*, is the perception of the conserved N-terminal part of flagellin (22-amino acids; flg22) covered by a host receptor-like kinase sensitive to flagellin, FLS2 (flagellin-sensitive-2 [28,29]). In many plants, FLS2-based recognition of pathogens represents one of the major early barriers to infection [2]. The rapid detection of flagellin most likely results from the conserved structure of flg22 as well as the properties of the flagellin epitope and glycosylation pattern [30,31]. Flagellin isolated from *Psp* and many other phytopathogenic pseudomonads is glycosylated in a conserved manner with two oligosaccharides, trisaccharides, and tetrasaccharides at a ratio of approximately 5:1 [32]. Some studies have also indicated that posttranslational flagellin modifications play a crucial role in the induction of HR by phytopathogenic pseudomonads [33]. Interestingly, this ligand–receptor interaction is likely still under strong evolutionary pressure, as experimental manipulations on non-*HB* models supported data showing that the plant flagellin receptor shows evolutionary plasticity [34] and that pathogenic bacteria can modify the epitope of flagellin to evade flagellin-mediated recognition without loss of motility [35]. However, whether the glycosylation state of *Psp* flagellin directly affects its recognition by common bean has not yet been experimentally addressed, making this an important open question for future studies on the early stages of *HB*.

If plant receptors perceive bacterial ligands such as flg22, early immune signaling is activated, including salicylic acid (SA)-dependent antibacterial responses [36]. In stomatal immunity, SA-dependent responses operate within a broader regulatory network involving reactive oxygen species (ROS) production, abscisic acid (ABA) signaling, mitogen-activated protein kinases (MAPKs) cascades, and ion fluxes and are further shaped by crosstalk between hormone pathways, particularly the antagonistic relationship between SA-dependent antibacterial defense and jasmonic acid (JA)-related signaling [37]. Therefore, to gain access to the apoplast, pathogenic pseudomonads must wait until favorable conditions take place or induce stomatal reopening [38]. This can be hypothetically achieved by altering plant signal transduction pathways and exploiting them for the purpose of invaders. Unfortunately, many aspects of host defense alterations during the initial phases of *Psp* infection have not yet been elucidated. Phytopathogenic pseudomonads can cause stomatal reopening in response to the jasmonic acid (JA) mimic–coronatine. This mechanism involves both canonical and noncanonical pathways [39]. However, this mode of action has been reported only for a few *P. syringae* pathovars, namely, pvs. *aesculi*, *atropurpurea*, *glycinea*, *maculicola*, *morsprunorum*, *oryzae*, and *tomato* [40,41,42], and is likely absent from *Psp* (Table 1). In all the studies on the comparative analysis of phytopathogenic strains, only one study reported the active production of coronatine (a phenotypic assay based on the reaction of plant tissues to artificial inoculation) by one *Psp* strain (which reported seven nonproducing strains at the same time [40]). Another common virulence factor that is synthesized by pseudomonads and responsible for stomatal opening through the depletion of SA signaling is syringolin, a toxic proteasome inhibitor [43]. Like coronatine, this compound is also not produced by *Psp* (Table 1). Nevertheless, one plausible but still experimentally unconfirmed scenario is that *Psp* may exploit auxin-related metabolism to facilitate stomatal reopening. Interestingly, the model strain of *Psp* (1448A) lacks a gene responsible for the enzymatic modification of auxin (*IaaL* [18]), and, to date, the available sequence data do not provide direct evidence that a full-length, functional *IaaL* gene is present in other *Psp* strains. *Psp* does not produce AvrRpt2, an effector known to induce auxin-dependent responses by altering the stability of key auxin transcriptional repressors [44]. However, the genome of the model strain *Psp* 1448A contains genes encoding other, non-Hop proteins potentially involved in auxin biosynthesis, namely, *IaaM* and *IaaH* [18,42]. Although hybridization data for strain INRAE CFBP 1390 were not fully consistent with the genomic data from *Psp* 1448A, the synthesis of indole-3-acetic acid (IAA) was confirmed using chromatography when *Psp* was grown in vitro on medium supplemented with L-tryptophan [45]. Taken together, these findings suggest that auxin-related traits may be relevant for some isolates, but they do not demonstrate manipulation of host auxin regulation or auxin-dependent stomatal opening or reopening in planta. This scenario still lacks direct in planta support, including evidence from appropriate loss-of-function *Psp* mutants. Moreover, other yet unknown mechanisms responsible for stomatal opening cannot be excluded.

Nevertheless, compared with the broad repertoire reported for phytopathogenic *Pseudomonas* species, only a subset of these non-effector extracellular virulence-associated factors has been documented for *Psp*; this should not be interpreted as evidence that the pathogen is functionally constrained but rather as an indication that its infection success is achieved through a specific combination of virulence-related traits (Table 1).

Another strongly environmentally driven mechanism that may indirectly facilitate host entry in some *Pseudomonas* species is frost injury mediated by ice nucleation proteins (INPs [52]). INPs are large, outer-membrane-associated proteins that increase the temperature threshold for heterogeneous ice nucleation, enabling ice formation at relatively high subzero temperatures [53]. Their repeat-rich domains form β-helices that increase the size of the water-ordering surface [53,54]. Ice nucleation-active bacteria can be detected on many herbaceous plant species, including bean [55]. However, functional INP production is doubtful in the reference *Psp* strain 1448A [18]. Although an *inaZ*-like locus is present in this strain, it is disrupted, making the production of a functional INP unlikely [18]. Consistently, *inaZ* has been used in *Psp* mainly as a highly sensitive heterologous reporter system [56]. Nevertheless, the possibility that mixed epiphytic microbiota composed of ice nucleation-active bacteria indirectly favor *Psp* entry by increasing frost-induced tissue damage cannot be ruled out.

The possible involvement of type three secretion system (T3SS) effectors further complicates the relationship between stomatal behavior and apoplast hydration. In another *P. syringae* pathosystem, AvrB was shown to promote stomatal opening through a pathway involving RIN4, AHA1, and jasmonate signaling [57]. In contrast, HopM1 and AvrE1 were shown to induce ABA-associated guard cell responses, stomatal closure, and the establishment of an aqueous apoplastic environment [58]. Therefore, effector-dependent manipulation of stomatal behavior may theoretically contribute either to host entry or to the stabilization of a hydrated apoplast, depending on the effector, host context, and infection stage. However, no *Psp* effector, including AvrB-like effectors or AvrE, has yet been directly demonstrated to reopen stomata or target guard cell signaling in common bean.

More globally, successful infection requires an efficient switch from an epiphytic/planktonic lifestyle to a pathogenic lifestyle, which is strongly associated with flagellar function (and, thus, motility). The expression of the *hrpL* gene (alternative sigma factor/master regulator of T3SS gene expression, which is associated with the downregulation of flagellar function) is strongly elevated when *Psp* is inoculated into plants, which increases the expression of selected effector genes and their carriers, i.e., the T3SS [59]. It seems that the molecular basis of this action is related to the detection of cell wall components [60], the pH value, and the local nutritional status [61]. Recent findings revealed that not all bacterial cells undergo a switch from a motile population to a pathogenic population, as *Psp* subpopulations show distinct patterns of division of labor [62].

**Figure 1:**
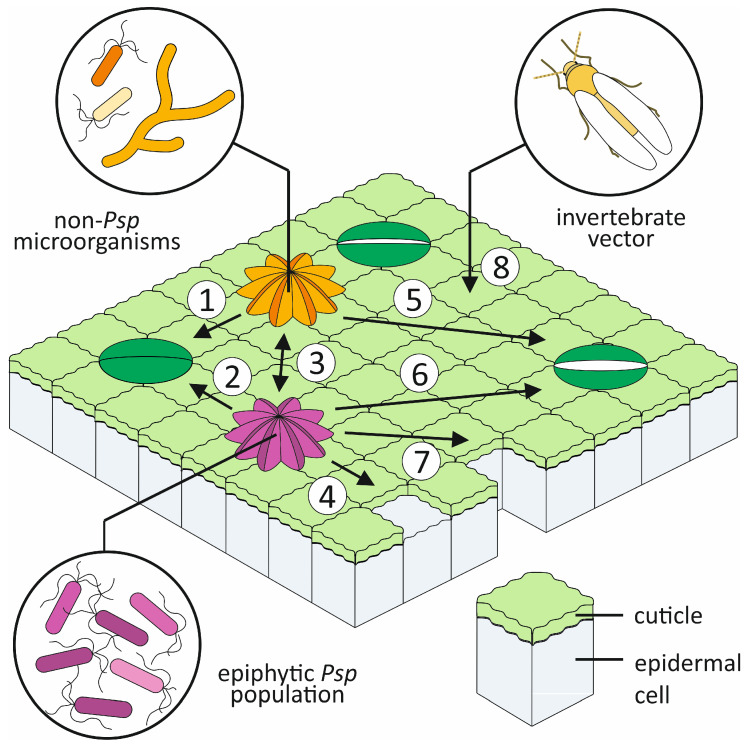
Simplified model of reported and hypothetical epiphytic interactions potentially contributing to the establishment of an in planta population of *Pseudomonas savastanoi* pv. *phaseolicola* (*Psp*). Numbering: 1—pathogenic and non-pathogenic non-*Psp* microorganisms may cause closure of plant stomata; 2—detection of *Psp* may cause stomatal closure; 3—communication based on quorum-sensing messengers and other metabolites of interacting epiphytic microorganisms (both pathogenic and non-pathogenic); 4—infection through disrupted cuticle (caused by abiotic and biotic stress factors); 5—opening of plant stomata by non-*Psp* epiphytic microorganisms (hypothesized); 6—opening of plant stomata by *Psp* to facilitate host entry (hypothesized); 7—infection through mechanically damaged tissues; 8—vector-based dispersal of *Psp* (hypothesized). The scheme is based on information presented in the sections where the figure is referenced.

Thus, early stages of a pathogenic lifestyle rely on the efficient relocation of resources from motility to shape a compatible habitat and its exploitation. In summary, at this stage, *Psp* appears to rely on opportunistic entry and an efficient switch between epiphytic and pathogenic lifestyles. Nevertheless, the number of factors and interactions that hypothetically affect successful entry is high (Figure 1), highlighting the need for further studies.

## 4. Shaping a Compatible Habitat and Its Exploitation

For hemibiotrophic pathogens, the apoplast is initially a conditionally hostile space, and successful colonization therefore requires active modification of this environment to suppress host immunity and establish a “safe space”. Thus, *Psp* must establish a permissive extracellular habitat and gain access to water and sufficient carbon (C), nitrogen (N), macro- and micronutrient sources to sustain replication within host tissues. In addition to exploiting nutrients already present in apoplastic fluid, *Psp* may also increase nutrient availability through various dedicated mechanisms, including toxins [46,47,48].

The accumulation of water in the apoplast due to the action of phytopathogenic bacteria, including pseudomonads, has been recently reviewed [63]. Although water-soaked lesions are typical of *HB* [2], the nuances of the molecular mechanism responsible for the formation of these lesions are better described for phytopathogenic pseudomonads other than *Psp*. For example, in *P. syringae* pv. *tomato*, the establishment of a watery apoplast is associated with AvrE/HopM1-dependent regulation of stomatal closure [63]. Whether this mechanism also operates in *Psp* remains unclear because, although an AvrE-encoding ORF is present in this pathovar, the HopM-encoding ORF is disrupted/truncated in strain 1448A [42]. Classic works on *HB* have suggested that extracellular polysaccharides (EPSs), particularly alginate and levan, may stabilize the hydrated state of the apoplast [64,65]. Nevertheless, more *Psp*-oriented studies are needed to determine whether EPS-mediated stabilization is sufficient to explain water accumulation in the apoplast during *HB*. Moreover, the intriguing question is whether competition occurs between epiphytic and internal populations of *Psp* based around the opening/reopening of stomata, as this has various hypothetical consequences for populations: while stomatal reopening facilitates migration into the leaf, it increases transpiration, hindering the formation of a watery apoplast.

Destabilization of host cell membranes and promotion of local nutrient leakage are among the possible ways to increase nutrient accessibility. Notably, *Psp* does not produce the cell membrane-damaging detergent-like toxins common to other phytopathogenic pseudomonads (Table 1). On the other hand, the HrpZ1 protein produced by *Psp*, an accessory protein implicated in effector translocation, has been shown to bind lipid bilayers and form ion-conducting pores in vitro [66]. This membrane-associated activity has led to the proposal that HrpZ1 may facilitate effector delivery/translocation and potentially contribute to nutrient leakage from host cells [67]. However, its quantitative contribution to nutrient release and apoplastic growth during *Psp* infection in planta remains unresolved. HrpZ1 can also be perceived by plants and trigger immune-associated responses [67,68]. Notably, intact HrpZ1 is required for pore formation, whereas the C-terminal fragment is sufficient to elicit plant immune responses [67].

Both C and N sources are crucial for successful pathogenesis. With respect to *Psp*, a limited number of studies have shown that the leaf apoplast provides a dynamic nutritional environment whose composition changes rapidly after infection and can support bacterial metabolism. In common bean, apoplastic concentrations of sucrose, several amino acids, and other metabolites increase within the first hours after inoculation, indicating that host infection is accompanied by a rapid redistribution of potentially accessible nutrients [69]. Importantly, however, *Psp* does not appear to exploit these compounds indiscriminately. Metabolic footprinting has shown that *Psp* preferentially utilizes malate, glucose, and glutamate, whereas other abundant apoplastic metabolites, such as citrate and GABA, are used only later, after preferred substrates become limiting [69]. In addition, different host-derived extracts, including leaf extracts, pod extracts, and apoplastic fluid, differentially regulate bacterial gene expression, including genes associated with aerobic metabolism, indicating rapid metabolic adaptation to the environment [21]. Notably, among the phytopathogenic pseudomonads compared thus far, *Psp* appears to have a relatively restricted nutrient utilization range, potentially highlighting a relatively high degree of specialization to specific host habitats [70].

*Psp* is able to exploit host metabolism to support its own nutritional requirements. The best known example is the production of phaseolotoxin (Table 1), an antimetabolite prototoxin that causes nonhost-specific chlorosis. Although this toxin is typical of *Psp* [3], some isolates are non-toxigenic [49]. Phaseolotoxin is composed of two moieties: the L-ornithyl-alanyl-homoarginine tripeptide and an inorganic moiety, the Nδ-N′-sulfodiaminophosphinyl group [41]. Once in plant tissues, phaseolotoxin is cleaved by host peptidases to generate Nδ-(N′-sulfodiaminophosphinyl)-L-ornithine (PSOrn [71]). The best-established mode of action of phaseolotoxin is the inhibition of host ornithine carbamoyltransferase/ornithine transcarbamylase (OCT; EC 2.1.3.3), resulting in the disruption of arginine biosynthesis [72,73]. Because ornithine is also connected to polyamine metabolism through ornithine decarboxylase (ODC; EC 4.1.1.17), phaseolotoxin-mediated perturbation of ornithine/arginine metabolism may also be associated with altered ODC activity [72]. However, OCT inhibition should be considered the primary and best-supported biochemical target of phaseolotoxin, whereas changes in ODC activity represent a related physiological consequence that requires more careful interpretation in the context of *Psp* infection. The resulting arginine deficiency contributes to functional impairment of host cells and is closely associated with the development of chlorosis [73]. Endogenous bacterial carbamoyl transferase (ArgK) is insensitive to this toxin [74]. As a result, the pathogen retains the ability to synthesize this amino acid for its own metabolism, including the production of phaseolotoxin and, hypothetically, the biosynthesis of ET [75]. The biosynthesis of this toxin is associated with the ability of the pathogen to cause disease symptoms and disseminate systemically in plant tissues [76,77]. Thus, by inhibiting host OCT and disrupting arginine biosynthesis while the pathogen remains protected by ArgK, phaseolotoxin acts not only as a symptom-inducing toxin but also as a metabolically strategic virulence factor that alters host primary metabolism in a manner favorable to *Psp* infection. Biosynthesis of phaseolotoxin is regulated by a complex web of environmental stimuli, such as temperature (whereby low temperature stimulates biosynthesis [77,78]), components of leaf tissues and apoplastic fluid [21], and oxidative stress [79]. Biosynthesis of phaseolotoxin is under the control of a multilayer mechanism. It is globally controlled by a two-component regulatory system (GacS/GacA [80]), which also regulates the expression of genes associated with the type six secretion system (T6SS, a conserved antibacterial secretion apparatus [81] of unknown ecological advantage in *Psp* [82]). Additionally, the Pbo cluster is responsible for the temperature-dependent regulation of phaseolotoxin biosynthesis [83]. Regulation based on oxidative stress (also involved in the regulation of pyoverdine biosynthesis) depends on OxyR protein, which also positively regulates the response of Pbo clusters [84]. These findings indicate that a multilayer mechanism is involved in the precise regulation of toxin biosynthesis. Interestingly, phaseolotoxin biosynthesis leads to the formation of a tripeptide byproduct (Cit-Ala-hArg), which can be utilized as a nontoxic, metabolically recoverable reservoir of citrulline (Cit) and the alanyl-homoarginine dipeptide (Ala-hArg) [85]. In this scenario, aminopeptidase-mediated cleavage of Cit-Ala-hArg may release Ala-hArg for re-entry into phaseolotoxin biosynthesis [85], whereas Cit can be redirected to further metabolism, most plausibly through the canonical arginine biosynthesis pathway.

In addition to hijacking host amino acid metabolism, many *Pseudomonas* species produce iron-chelating metabolites, namely, siderophores (Table 1). Under conditions of iron starvation, *Psp* produces those molecules for active uptake of iron (see review [86] for more details of pyoverdine chemistry and biology in pseudomonads). Although genetic studies have shown the presence of genes associated with the production of three siderophores (pyoverdine, achromobactin, and yersiniabactin), only two of them seem to be produced in detectable amounts: pyoverdine and achromobactin [51]. The expression of genes involved in the synthesis of siderophores was shown to be upregulated by cool temperature (18 °C), which is the optimal condition for *Psp* virulence [78]. Recent findings have shown that the synthesis of pyoverdine is coregulated with the biosynthesis of phaseolotoxin in an OxyR-dependent manner [84]. Efficient scavenging of available iron (which is in short supply in the apoplast of healthy bean plants, ca. 0.4 ppm [69]) may increase the survival rate of bacteria, but the picture is complicated. Even triple knockout mutants of *Psp* unable to produce pyoverdine, achromobactin, and yersiniabactin are still able to survive and display water-soaked spots in the pod-inoculation test [51]. On the other hand, it has been shown that pyoverdine is an intrinsic virulence factor for *P. syringae* pv. *tabaci* [87]. Although there is no direct evidence that *Psp* uses xenosiderophores in planta, this possibility can be considered an untested ecological hypothesis. This finding is conceptually consistent with the plant–bacterial supernetwork framework [88] and the broader observation that xenosiderophore use can contribute to microbial iron acquisition in some systems [89]. However, whether *Psp* can recognize or benefit from iron chelators produced by other microorganisms or the host has not been experimentally tested. Thus, xenosiderophore use should not be treated as an established component of *Psp* iron acquisition but as a hypothesis-generating question for future studies.

Data on the acquisition of other crucial elements, e.g., manganese (Mn), zinc (Zn), and copper (Cu), by *Psp* are very scarce and can be extrapolated only from studies on other pseudomonads, which indicates that *HB* can be a plentiful study area in the near future. It must, however, be noted that highly elevated Cu concentrations, in the range of ca. 25–50 ppm (approximately twofold higher than expected under apoplastic conditions), are likely to present a physiological challenge for Cu-sensitive *Psp* isolates [90]. Nevertheless, most tested *Psp* isolates were shown to be Cu-tolerant [90].

Therefore, shaping the compatible habitat by *Psp* is based on metabolic engineering of the apoplast, with phaseolotoxin as a major yet described factor, acting as both a symptom-inducing toxin and a resource-redirecting virulence factor.

## 5. Evasion of the Host Immune Response

Evasion of the host response plays a crucial role in pathogen survival. Thus, phytopathogenic pseudomonads have evolved escape strategies that are based on many effector proteins. The molecular targets of given effectors produced by phytopathogenic pseudomonads have been extensively studied (see review [91]). The role of many effectors from the repertoire of *Psp* has also been analyzed [92]. The literature on the role of a given effector in pathogenesis is rich; thus, although these data need to be reviewed in the near future, the following paragraphs are aimed at (not only) effector-based rearrangements in *Psp*, allowing evasion of the host response.

Stressful environments may induce chromosome rearrangements, resulting in *Psp* hiding in plain sight. Frequent passages of bacteria (*Psp* 1302A) between resistant bean plants (‘Tendergreen’) resulted in the selection of bacterial cells that were able to cause disease symptoms (75% of isolated colonies), whereas fewer HR-inducing bacterial cells were present (25% of isolated colonies [93]). This change was associated with the loss of a large chromosomal region (106 kb) containing the *hopAR1* (former name *avrPphB*) gene, named PPHGI-1. *Psp* 1302A can colonize a wide range of common bean cultivars, and this ability is connected to the loss of the PPHGI-1 region [93,94]. Moreover, PPHGI-1 can be transferred between strains of *Psp* in planta [95], and, after excision from the chromosome, it becomes a circular episome (capable of replication), which results in a great reduction in *hopAR1* expression. Other studies have also indicated that PPHGI-1 can be permanently lost in some strains during passaging in resistant cultivars, but a small percentage of the bacterial population always seems to retain PPHGI-1 [96]. As indicated in further studies, this phenomenon might result from increasing amounts of hydrogen sulfide (H_2_S) produced during the plant response [97]. Such a mode of action indicates a sophisticated mechanism of evading recognition on the basis of ‘gene banking’ but not necessarily total and persistent loss of effector genes.

Modifications of gene sequences were also listed as mechanisms for reducing host HR formation. Studies on the distribution of *hopX1* alleles (formerly named *avrPphE*) revealed small-scale changes (single base-pair substitutions) in *Psp* races 1, 3, 6, and 9 as well as insertions (104 bp containing border 12 bp inverted repeats) in race 8, which are not able to induce HR [98]. Similarly, another effector, HopQ1, is under evolutionary pressure, and its recognition by the host relies on the sequence of this effector [99]. Additionally, the expression of another effector, HopR1, masks HopQ1, reducing the recognition of pathogenic bacteria by the host [99]. Nevertheless, the picture of effector expression patterns seems to be much more complicated, as it was recently proposed that *Psp* effectors are likely epigenetically controlled. The methylation profile of DNA has been shown to regulate the function of the T3SS and is dependent on the growth phase of *Psp* and, hypothetically, environmental conditions [100]. Another source of variation is the specific activity of *Psp* subpopulations. Recent findings have shown that *Psp* subpopulations living in planta undergo separate adjustments, whereby some subpopulations suppress host immunity through the T3SS, whereas others (composed of motile flagellum-expressing individuals) migrate from primarily infected tissue before necrosis takes place [62].

Some hormonal compounds, namely, ET, and effectors altering its biosynthesis can also be considered important components of pathogenesis. In *P. syringae*, the HopAF1 effector suppresses the biosynthesis of ET by the host, altering its immune response [101]. In contrast to this observation, although *Psp* isolated from common bean does not produce ET (Table 1), strains isolated from another legume plant species, kudzu (*Pueraria lobata* (Willd.) Ohwi), produce significant amounts of this hormone [50,102]. Interestingly, ET is known to play a role in plant pathogenesis [103], but for *Psp*, ethylene-forming enzyme (*efe*)-negative kudzu-derived isolates did not have a lower multiplication ability than the wild-type strain did [104]. Further molecular studies indicated that in the best-characterized *Psp* strain (1448A), an ORF for the *efe* gene is absent [42]. These findings are in agreement with the concept of two evolutionarily separated *Psp* lines, which are associated with common bean and kudzu [105]. Interestingly, arginine is involved in ET biosynthesis in kudzu-isolated *Psp* (strain PK2 [106]). Nevertheless, the PK2 strain isolated from kudzu was shown to be phaseolotoxin negative [104]. Therefore, although the link between arginine metabolism and ET/phaseolotoxin synthesis still needs to be elucidated, the role of ET in *Psp* pathogenesis seems to be even more enigmatic.

*Psp* is also able to avoid the toxin action of phytoalexins, which are low-mass specialized plant metabolites produced during infection. For example, two isoflavonoid phytoalexins of bean (genistein and daidzein) are efficiently degraded enzymatically by *Psp*; additionally, *Psp* stimulated by these compounds synthesizes a set of cyclic dipeptides of enigmatic function [107]. On the other hand, another phytoalexin, phaseollin, which is toxic to *Psp* in vitro [108], halts the growth of the *Psp* population in planta [109] and accumulates in host plants during infection. The accumulation of this antimicrobial agent was 15 times lower in the compatible pathosystem of strain 1448A (race 6) and ‘Sehiradi-90’ bean plants (susceptible) than in the incompatible pathosystem of strain 1302A (race 3) and the same cultivar (HR [109]), which can be attributed to the effector repertoire of *Psp* [110]. In contrast to genistein and daidzein, there is no published evidence showing that *Psp* is able to degrade phaseollin enzymatically. The mechanism underlying this apparent selectivity toward different bean isoflavonoids remains unresolved.

Not all traits of *Psp* are tuned to avoid detection by the host, but their role is elusive. There is a class of low-molecular-weight molecules triggering HR in plants of still unclear significance: syringolides (Table 1). They are acyl glycosides, the synthesis of which can be detected in phytopathogenic *Pseudomonas* species and several other phytopathogenic bacteria [111,112]. In *Psp*, it is governed by the *avrD* gene located on the native *Psp* plasmid [113]. The syringolide product from the enzymatic reaction of the protein encoded by *avrD* from *Psp* is nevertheless structurally different from that of *avrD* from *P. syringae* pv. *tomato* [111,114]. Although events that take place in plants after syringolide treatment are known mostly for *Glycine max* (L.) Merr. [115], trackable records of more recent studies addressing the adaptive and ecological role of these molecules for phytopathogenic *Pseudomonas* species, including *Psp*, are lacking. This leaves unresolved why *Psp* maintains the capacity to synthesize HR-triggering molecules despite being able to establish compatible infections. Whether this apparent paradox results from temporal or spatial separation between syringolide production and T3SS-dependent immune suppression or another yet unknown function of syringolides remains unknown for *Psp*.

In summary, the current understanding of the evasion of the host immune response by *Psp* is based on effectors and their expression. Further studies on the combined role of effectors, epigenetic regulation of their expression, ecological advantage of ET synthesis, ability to degrade phytoalexins, and role of syringolides/other small molecules will shed light on crucial aspects of *Psp* biology related to evasion of the host response.

## 6. Multiplication

When pathogenic bacteria are able to find a permissive habitat and evade the host immune response, they can multiply when there are no other obstacles. Time–course growth curves of *Psp* are available only for a few bean cultivars differing in susceptibility to *HB*, including ‘Borlotto di Vigevano’ and ‘Saluggia’. Under controlled environmental conditions (23/16 °C, 12/12 h day/night thermophotoperiod) and inoculation conditions, in which approximately 0.1 cm^3^ of bacterial suspension was retained by the leaf and more than 99% infiltrated into the mesophyll, bacterial population growth was described as comprising an early phase of increase, a phase of decelerating growth, and a stationary phase [116]. In these experiments, early population increase could be approximated by exponential growth for approximately 3 days in ‘Borlotto di Vigevano’ and 5 days in ‘Saluggia’ [116]. During the 24–48 h interval after inoculation, the apparent net population doubling times were approximately 5.8–6.2 h in ‘Borlotto di Vigevano’ and 8.7–9.1 h in ‘Saluggia’, depending on the inoculum dose [116]. However, these results may not precisely reflect field-representative doubling times.

The size of total and epiphytic *Psp* populations depends on environmental conditions. Rainfall is followed by an increase in population size, whereby a lack of symptoms supports the hypothesis that the availability of water affects epiphytic populations [22]. The availability of water to epiphytic *Psp* populations is also regulated by the microclimate, which can result from the cropping system. For example, compared with monocultures, the intercropping of beans with maize favors an increase in the size of internal and epiphytic populations (‘Canadian Wonder’); taking into account the low ability of *Psp* to survive in/on maize leaves, these differences can be attributed to the slow drying of bean leaf surfaces after rainfall in the intercropping system [117]. Additionally, multiplication depends on the host’s resistance to *HB*. Classic works have shown that the total size of the population (from inoculation) varies among bean cultivars [118]. Additionally, spray inoculation of bean cultivars differing in *HB* resistance yields epiphytic/total *Psp* populations of various sizes, whereby the sizes of epiphytic and total *Psp* populations in the susceptible cultivar (‘Charlevoix’) were 2.5–3.4 and 1.6–2.8 orders of magnitude greater, respectively, than those in resistant cultivars (‘Montacalm’ and ‘Seafarer’; recalculated using data from the literature [119]). These findings indicate that resistance to *HB* in beans is also associated with traits allowing the survival of epiphytic populations. It should be noted that direct quantitative studies of *Psp* multiplication in planta remain relatively scarce, and many foundational data on population growth, interdivision time, and cultivar-dependent multiplication still come from classical experiments that have not been superseded. More recent work has not replaced these data with new bulk growth curves but has shifted attention toward other, mostly molecular and population-level determinants of infection success.

Although data on the molecular aspects underlying successful multiplication are fragmentary, the programmed traits of *Psp* affect its ability to multiply in planta. For example, selected effectors contribute to the ability of *Psp* to multiply. Compared with those of the wild-type 1448A *Psp* strain, populations of some single-knockout mutants have been shown to have a weaker ability to grow in the *HB*-susceptible bean cultivar (‘Canadian Wonder’ [92]). However, mixed infections can facilitate the proliferation and local spread of non-pathogenic variants when they occur in close proximity to pathogenic bacteria [120]. Recent single-cell analyses further indicated that multiplication in planta should not be interpreted as a uniform process in which all bacterial cells contribute equally to population growth. It has been shown that *Psp* 1448A forms phenotypically heterogeneous subpopulations differing in T3SS and flagellar expression during the colonization of beans [62]. The expression of the T3SS imposes a clear growth cost, as the constitutive expression of *hrpL* reduces bacterial competitiveness in planta [62]. Flagellar expression also has a growth cost, although this cost is more evident at the single-cell level: the expression of flagellin-encoding *fliC* is negatively correlated with the growth rate [62]. The strongest growth advantage was observed in the mutant, in which both T3SS activation and flagellin production were disrupted [62]. These findings suggest that multiplication is shaped by costly phenotypic specialization rather than by uniform activation of all virulence- and dispersal-related traits. However, the absence of T3SS and flagellar expression does not necessarily imply faster multiplication. Double *rsmA*-*rsmE* mutants of *Psp* 1448A, with RsmA and RsmE acting as global post-transcriptional regulators, have reduced expression of *hrpL*, *hrpA* (encoding the Hrp pilus subunit), and *fliC* but are nevertheless impaired in terms of symptom development, in planta growth, induction of the HR in tobacco, motility, timely phaseolotoxin biosynthesis, and carbon-source utilization on carbon source-limiting media [121]. Thus, the growth cost of T3SS and flagellar expression must be interpreted within the broader regulatory context: successful multiplication requires not only avoiding unnecessary energy burdens but also maintaining the regulatory capacity to suppress host immunity, exploit limited carbon sources, and coordinate the traits required for apoplastic colonization.

Taking into account the establishment of permissive habitat, the transition to population increase in planta is unlikely to depend simply on the presence/absence of nutrients in the apoplast. Data on apoplastic metabolite profiles reported in the literature [69] suggest that the compatible interaction of ‘Tendergreen’ bean with *Psp* RJ3 and the incompatible interaction with *Psp* 1302A diverge strongly by approximately 8–10 h after inoculation, a period that coincides with the beginning of the maintenance of bacterial multiplication by RJ3 and the restriction of 1302A [69]. This divergence is characterized not by a globally nutrient-rich apoplast (i.e., more nutrient-rich than the water-infiltrated control) but by selective differences in metabolite retention and accumulation [69]. In particular, compared with 1302A apoplasts, RJ3-associated apoplasts exhibit strong accumulation of gluconate and pyruvate and partial retention of citrate and several amino acids, whereas malate, glucose, and glutamate are depleted in both interactions [69]. These observations support the concept that successful multiplication may depend on the establishment of a permissive metabolic habitat rather than on simple nonspecific host cell damage or general nutrient leakage. For some metabolites, however, it cannot be stated now whether changes in their availability in the apoplast are host- or pathogen-driven. Accordingly, whether this set of metabolites contributes directly to the maintenance of the exponential growth of *Psp* remains unknown.

Stress caused by agrotechnical treatments, e.g., the use of antibiotics and biocontrol compounds, also reduces the ability of *Psp* to multiply. Nevertheless, they are inefficient at halting the reestablishment of populations from persisters when used alone, as culturable fractions from 1448A *Psp* cultures treated with streptomycin and tailocin are still able to infect host plants (‘Kentucky Wonder’ [122]). However, the joint stress caused by both agents completely stops multiplication [122].

In summary, although the multiplication of *Psp* is a crucial component of *HB* epidemics, data pertaining directly to events allowing/halting the growth of population size are less abundant than data on molecular events and mechanisms associated with host colonization and the shaping of compatible habitat. Further exploration of this topic is thus highly important for field practice.

## 7. Spread to Another (Not Necessarily) Compatible Host

Finally, efficient spread is the last stage resulting from successful multiplication, nested strongly in the environment and having weighty epidemiological consequences. The local and long-distance spread of bacteria can be achieved by infested seeds, passive transfer with water (rain splash and irrigation water), physical contact with living plants, plant debris and agrotechnical equipment, or transition by pests.

Seeds of *Psp* hosts can be contaminated (surface contamination) or infected (internal infection); however, in contrast to the seeds of susceptible bean cultivars, culturable *Psp* was not recovered from the seeds of tolerant cultivars [123]. Internal infection is very advantageous for *Psp*, as it allows it to persist in propagules (making it invulnerable to surface decontamination); in such cases, *Psp* migrates to developing seeds through the funiculus [124]. Interestingly, the fine-scale localization of viable bacteria within seed anatomical structures remains unresolved. In general, the seedborne nature of *HB*, whereby infected seeds do not necessarily show disease symptoms [2,123], but even a few infected seeds are sufficient to initiate a general epidemic under favorable conditions [125], is perceived as a major characteristic of this disease that allows long-distance transmission of the pathogen and makes its control difficult.

Another crucial component of disease spread is the availability of water, which acts as the primary medium enabling short-distance (field scale) *Psp* dispersal. Water, particularly precipitation, rain splashing, and irrigation water, is a major vector facilitating the dissemination of foliar bacterial pathogens across spatial scales [126,127].

**Figure 2:**
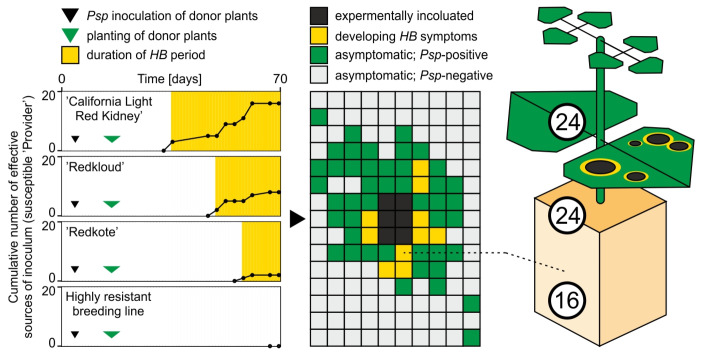
Contribution of cultivar resistance to halo blight (*HB*) epidemics, local-scale dispersal of *Pseudomonas savastanoi* pv. *phaseolicola* (*Psp*), and the potential survival of *Psp* in field reservoirs. The left-side panel represents the contribution of cultivar resistance/tolerance of donor *Psp*-infected plants to disease outbreak in a susceptible cultivar (newly redrawn representation based on data from the literature [128]). The middle panel represents the local spread of *HB* symptoms and *Psp* one month after experimental inoculation (newly redrawn representation based on data from the literature [22], licensed under CC BY 4.0; no original file was reused). The right-side panel represents the maximum reported survival time of bacteria (numbers indicate time in weeks) on the basis of data from the literature [129,130].

The speed and scale of disease spread (Figure 2) are modified by the resistance/tolerance level of the cultivar [128], and when favorable environmental conditions occur, relatively fast progression of *Psp* spread can be observed [22].

There are also several other factors related to agronomic treatments that facilitate *Psp* transmission. For example, *Psp* isolated from plant debris in eastern Africa (races 1, 2, and 3) was able to infect an *HB*-resistant bean cultivar (‘Edmund’) and survive on bean debris for up to six months, but an increasing host resistance level, as well as an increasing burial depth, reduced survival time [130]. Similar results were reported in a study in which *Psp* was able to survive on nylon netting for up to six weeks both under and on the soil surface [129]. Additionally, on infected plant debris, bacteria survived for 11 weeks when buried and even for 20 weeks on the soil surface [129]. These findings indicate that plant debris or agrotechnical equipment remaining in the field can be reservoirs of viable pathogens during the season (Figure 2).

It can also be hypothesized that *Psp* transmission is partially governed by insects (Figure 1). A study on oral inoculation with artificial diets containing *Psp* on aphids (*Acyrthosiphon pisum*) and whiteflies (*Bemisia tabaci*) revealed that *Psp* significantly reduced the population of aphids (by 35–44%) and whiteflies (by 20–35% [131]). Even if direct insect-mediated transmission of *Psp* remains unconfirmed, such reductions in insect populations may still be ecologically relevant because changes in the abundance of potential vectors or herbivores could indirectly affect local disease-spread dynamics. The question of whether insects that survived feeding on plants with a considerable presence of *Psp* could be responsible for *Psp* transmission between plants remains unanswered, as the bacteria were not reisolated from the insects in this study. Evidence from studies on other *P. syringae* pathovars suggests that this, however, can be the case [132,133], indicating the need for further studies on the insect-assisted transmission of *Psp*.

Since its first description, *HB* has been reported on all continents except Antarctica [2,124]. The worldwide distribution of *Psp* is associated with main bean-producing areas [134], suggesting that the list of hosts can therefore depend on geographical location and local flora. *Psp* was listed as pathogenic (Table 2) for many species (taxonomy according to the World Flora Online [135]) from the *Fabaceae* family [10,50,130,136,137,138,139,140,141,142]. Predominantly, *Psp* can infect a large number of species belonging to the *Phaseoleae* tribe, including *Phaseolus* species [2]. In addition to *Phaseolus* species, natural host species, namely, *Pueraria lobata* (Willd.) Ohwi, *Neonotonia wightii* (Wight & Arn.) J.A.Lackey, and *Vigna radiata* (L.) R.Wilczek, seem to be the most studied. The last of those species was recently indicated to be strongly threatened by *Psp* in Asia and Australia [140,141]. Additionally, epiphytic *Psp* was reported from nonlegumes, and, interestingly, these isolates are pathogenic to beans [137,142]. However, the epidemiological significance of isolates from non-bean hosts for bean cultivation has still not been fully determined. Although studies concerning the survival and multiplication of *Psp* on different artificial hosts are available [118], there are only a few reports of *Psp* colonization/infestation on non-bean species under field conditions. Accordingly, the seedborne nature of *Psp* in non-bean species has not been elucidated to date. Furthermore, many nonleguminous weeds and cultivated plant species are listed as alternative hosts. Thus, although regulatory authorities provide *Phaseolus vulgaris* as a major host for *Psp*, the list of potential hosts and plant reservoirs is much richer (Table 2). Notably, many of these species are annual plants, but the ability of *Psp* to survive in plant debris (described above) can hypothetically influence disease outbreaks annually. Comparative studies have suggested that variations in effector repertoires, including *hopC1* and *hopM1*, may contribute to host specialization [42].

In summary, alternative hosts and epiphytic reservoirs may contribute to local persistence and disease outbreaks, but their quantitative importance for *HB* outbreaks remains insufficiently resolved. These findings also indicate the urgent need for complex studies on the factors contributing to the species-to-species transfer of *Psp*.

## 8. An Open Question: Can We Stop This Disease and, If So, How?

It is widely held that diseases caused by phytopathogenic pseudomonads are among the most challenging to control. Therefore, available and emerging control strategies can be interpreted as interventions targeting different stages of the infection cycle: copper-based treatments mainly affect epiphytic populations before entry, and resistance breeding limits immune evasion and internal colonization, whereas antibiotics, tailocins, and biological control agents are expected to act mostly after bacterial multiplication has begun.

The best way to control *HB* is likely to involve the planting of resistant or tolerant bean cultivars. In agreement, new cultivars and germplasm lines have developed from time to time. For example, the pinto class common bean germplasm line US14HBR6 (with acceptable market parameters), possessing two recessive genes causing resistance to *Psp* race 6 (globally prevalent and broadly virulent) registered in 2014, can be treated as a breakthrough in the development of *HB*-resistant cultivars [143]. These findings indicate that the development of more cultivars resistant to race 6 and other races can be expected in the near future. On the other hand, many currently cultivated bean lines are rather susceptible to *HB*. For example, fewer than 5% of the 146 common bean cultivars indicated in the CRSP (Bean/Cowpea Collaborative Research Support Project) list of bean varieties registered between 1980 and 2010 are *HB*-tolerant or *HB*-resistant [144]. Finding tolerance or resistance to *HB* is not always easy. Resistance genes (responsible for race-specific resistance) were present in 49.4% of the 1048 tested common bean accessions [145]. However, small-scale local screening of bean accessions for resistance to *HB* revealed only one genotype (among the 38 tested) that could be a promising candidate for breeding studies (data for commercial bean fields in Turkey [146]). Resistance to *HB* seems to be associated with an adequate repertoire of host receptors. Quantitative trait loci (QTLs) studies on resistance to *HB* link resistance to *Psp* races 3, 4, and 5 with a cluster located on chromosome 2 (Pv02), a cluster of nine genes located within a 3.01 Mb region [147]. Furthermore, resistance to *Psp* observed in ‘UI3’ common bean was proposed to be a result of immune response activation covered by combinations of resistance proteins, which suggests that the ‘guard model’ is an important mechanism involved in bean resistance to *HB* [12]. These findings are consistent with the observation that genes within the Co-4 locus, including COK-4-related receptor-like kinases, are transcriptionally regulated after flg22 treatment or challenge with *Psp*, suggesting a possible role in basal immune responses rather than directly proven resistance to *HB* [148]. Similar concepts have been proposed in other studies, such as four significant QTLs in the linkage group at Pv04 and Pv06, and it has been proposed that resistance associated with those QTLs can be associated with 16 candidate genes of receptor proteins containing leucine-rich repeats (LRRs [149]). Further studies on bean accessions resistant to *Psp* race 6 revealed that two QTLs, HB4.2 on Pv04 and HB5.1 on Pv05, are associated with resistance [150]. Interestingly, whereas HB5.1 confers resistance to *Psp* race 6 only, HB4.2 also confers resistance to all other races of *Psp* [150]. Recent studies not only confirmed these two QTLs but also supported evidence for five others (HB1.1, HB1.2, HB3.1, HB8.1, and HB10.1) [151]. These QTL regions contain candidate genes encoding nucleotide-binding leucine-rich repeat (NLR)-type proteins, LRR-containing proteins, malectin-like proteins, or receptor-like kinases, among others [147,148,149,150,151]; however, no cognate resistance protein within these QTL regions has yet been assigned to the recognition of specific *Psp* effectors. Clearer effector-linked relationships are known for some race-specific *Pse* loci, including *hopF1*–*Pse-1*, *hopX1*–*Pse-2*, and *hopAR1*–*Pse-3* [152], but analogous links remain unresolved for *Pse-4*, the poorly resolved *Pse-5*, *Pse-6*, and the QTL regions discussed here. Importantly, some classical *Pse* loci are genetically connected with broader *HB* resistance regions; for example, *Pse-1* and *Pse-2* are located on Pv10, where HB10.1 was also detected; *Pse-3* co-segregates with the resistance locus on Pv02; and *Pse-6* has been mapped to the HB4.2 region on Pv04 [147,150,151]. The unresolved nature of this effector–resistance gene connection is particularly evident for the HB4.2/*Pse-6* region. Although this interval contains a cluster of NLR protein-encoding candidate genes, it also includes a polymorphic gene encoding an RNA-binding protein implicated in the post-transcriptional regulation of plant immunity [150]. Thus, even in a region genetically linked with race-specific *Pse-6* alleles, whether resistance is mediated by one or more NLR proteins, an RNA-binding protein, or a functional combination of receptor and non-receptor components remains unresolved. Nevertheless, all these discoveries bring us closer to achieving the ambitious goals of molecular breeding aimed at facilitating tolerance or resistance against *HB*. This also raises questions of whether it will be possible to develop varieties resistant to all *Psp* races, whether these varieties will possess desirable market traits, and whether *Psp* is a sufficiently adaptable bacterium to evade fully engineered resistance to *HB* in the future. To date, most resistance genes and QTLs used or proposed for *HB* control appear to act after bacteria have already passed external protective barriers, primarily by limiting immune evasion. It cannot be ruled out that other stages of infection could be stopped by the host genetic repertoire, which can be achieved by both breeding programs and genetic engineering.

One of the most popular recommendations for the control of *HB* is the use of copper as a preventative agent that limits epiphytic populations and thereby reduces the probability of successful leaf entry and colonization. Nevertheless, copper-based formulations are not very efficient for the control of this pathogen. For example, nearly 80% of 35 isolates recovered from commercial snap bean fields in Florida have been shown to be copper-resistant in in vitro tests [90]. Although formulations containing mancozeb (dithiocarbamate fungicide) as a secondary agent improved the effectiveness of copper in greenhouse experiments, a complete reduction in disease severity was not achieved. Mancozeb has been reported to have antibacterial activity against *P. syringae* pv. *tomato* under in vitro conditions [153], but direct evidence for a comparable effect on *Psp* is limited. In conclusion, the use of copper formulations is not sufficient for the successful management of *HB*. The use of other, more aggressive agents, such as antibiotics (e.g., streptomycin), although successful at halting multiplication, poses risks to the environment and is banned in multiple countries. However, it must be noted that tailocin increases the efficiency of streptomycin [122]. These findings highlight the need to screen alternative chemical methods for the control of *HB*. Recent reports have indicated that bacterial and volatile organic compound-based formulations may be promising for achieving this goal [154].

## 9. Future Perspectives and Conclusions

### 9.1. Future Perspectives

The main limitation in understanding *Psp* infection biology is not the absence of information on individual virulence-associated traits but the difficulty in interpreting these traits across different scales. Some conclusions can be drawn directly from in planta studies on *HB*, whereas others are inferred from the genomic potential of *Psp* and in vitro assays interpreted by extrapolation from related pathovars. This involves processes linking host entry, shaping of habitat, immune suppression, multiplication, and spread. In such cases, the key question is not only whether *Psp* possesses a given trait or is able to realize a given scenario but also when it is deployed, under which environmental conditions, in which bacterial subpopulation, under which host genetic background, and with what consequence for disease development. Apparent contradictions in the literature, such as rare reports of coronatine production, strain-dependent auxin and ethylene-related traits, and the weak virulence impact of siderophore loss despite clear iron-scavenging capacity, should be treated not as minor inconsistencies but as indicators that several virulence-associated traits are strain- and/or condition-dependent.

This issue is important for *HB* control. The most relevant gaps are not necessarily the most complex ones but those that define where the infection cycle can be interrupted. If opportunistic entry is the dominant bottleneck of disease initiation, management should focus on inoculum pressure, surface survival, and weather-dependent risk. Conversely, to the extent that *Psp* actively alters stomatal behavior or apoplastic conditions, early infection becomes a more specific and challenging target for crop protection. Similarly, if alternative hosts and symptomless reservoirs, including weed species in bean production systems, as well as invertebrate vectors, contribute substantially to *Psp* persistence, then control based only on seed health, copper treatments, or resistant cultivars may remain incomplete.

Further studies should therefore move beyond testing virulence factors as isolated units and address how these factors function across infection stages. Integrating molecular genetics, plant physiology, microbial ecology, and epidemiology will be necessary to distinguish mechanisms that are merely intriguing from those that are actually limiting for *HB* development under field conditions. Accordingly, stage-specific questions (Table 3) are intended as priorities for connecting the mechanistic understanding of *Psp* infection with disease management.

### 9.2. Conclusions

The plethora of results arising from studies on isolated virulence factors of *Psp* supports the construction of an operational stage-based infection framework for *P. savastanoi* pv. *phaseolicola*. This framework not only identifies major knowledge gaps but also helps position individual virulence-associated traits within specific stages of the infection cycle, including colonization, compatible habitat establishment, immune evasion, multiplication, and spread.

Like other well-characterized phytopathogenic *Pseudomonas* pathovars, such as *P. syringae* pv. *tomato* and *P. syringae* pv. *syringae*, *Psp* relies on traits that support the abovementioned stages of the infection cycle. However, the configuration of traits differs among these phytopathogenic strains, making *Psp* distinct. Other pathovars rely on the reopening of stomata by coronatine and the disruption of host physiology by the T3SS (*P. syringae* pv. *tomato*), or on syringolin-based toxicity and lipodepsipeptide-/INP-based cell damage (*P. syringae* pv. *syringae*). In contrast, *Psp* appears to colonize host tissues largely opportunistically, has a relatively limited repertoire of membrane-damaging extracellular factors, shapes host physiology mainly through phaseolotoxin-mediated disruption of primary metabolism, and manipulates host immune response in T3SS-dependent manner.

Thus, the narrower repertoire of extracellular virulence-associated factors does not make *Psp* a pathogen with a simpler infection strategy but rather one with a differently coordinated strategy. A central take-home message of this review is that *Psp* infection biology should not be reduced to isolated virulence traits. The biological significance of each trait depends on the infection stage, strain background, host genotype, bacterial subpopulation, and environmental context in which it operates. Ultimately, this stage-based interpretation helps distinguish mechanisms directly supported for *Psp* from hypotheses extrapolated from related pathosystems. This distinction is essential for identifying which stages of halo blight development can be most effectively targeted by future mechanistic studies and disease management strategies.

## Figures and Tables

**Table 1:** Selected extracellular virulence-associated factors, siderophores, and elicitors reported for *Pseudomonas savastanoi* pv. *phaseolicola* in contrast to other known phytopathogenic *Pseudomonas* species.

Virulence Factor	Produced by *Psp*?	Structure	Role
Antimetabolite toxins			
Phaseolotoxin	+ ^1^	Sulfodiaminophosphinyl peptide	Ornithine carbamoyltransferase (EC 2.1.3.3) inhibitor
Tabtoxin	−	β-lactam	Glutamine synthetase (EC 6.3.1.2) inhibitor
Mangotoxin	−	Oligopeptide	Ornithine N-acetyltransferase (EC 2.3.1.2.5) inhibitor
Rhizobitoxine	−	Vinylglycine derivative (cystathionine analog)	Cystathionine β-lyase (EC 4.4.1.8) inhibitor
Detergent-like toxins			
Syringomycins	−	Cyclic lipodepsinonapeptide	Detergent, pore formation, antimicrobial
Syringotoxins	−	Cyclic lipodepsinonapeptide	Detergent, pore formation, antimicrobial
Syringostatins	−	Cyclic lipodepsinonapeptide	Detergent, pore formation, antimicrobial
Pseudomycins	−	Cyclic lipodepsinonapeptide	Detergent, pore formation, antimicrobial
Tolaasins	−	Cyclic lipodepsinonapeptide	Detergent, pore formation, antimicrobial
Viscosin	−	Cyclic lipodepsipeptide	Detergent, pore formation, antimicrobial
Cormycin	−	Cyclic lipodepsipeptide	Detergent, pore formation, antimicrobial
Corpeptin	−	Cyclic lipodepsipeptide	Detergent, pore formation, antimicrobial
Syringopeptins	−	Cyclic lipodepsipeptide	Detergent, pore formation, antimicrobial
Fuscopeptins	−	Cyclic lipodepsipeptide	Detergent, pore formation, antimicrobial
Amphisins	−	Cyclic lipodepsipeptide	Detergent, pore formation, antimicrobial
Other toxins			
Persicomycin	−	Fatty acid	n.d.
Tagetitoxin	−	Sulfur-containing bibyclic compound	RNA polymerase III (EC 2.7.7.6) inhibitor
Tropolone	−	Non-benzoic aromatic compound	Polyphenol oxidase (EC 1.14.18.1) inhibitor
Syringolin	−	Cyclic peptide	Proteasome inhibitor
Phytohormones and their mimics			
Ethylene	+ ^2^	Alkene	Alteration of host defense pathways
Indole-3-acetic acid	+ ^3^	Auxin	Alteration of host defense pathways
Coronatine	− ^4^	Polyketide (JA-Ile mimic)	Alteration of host defense pathways
Iron chelators			
Yersiniabactin	− ^5^	Siderophore	Iron-scavenging
Pyoverdin	+	Siderophore	Iron-scavenging
Achromobactin	+	Siderophore	Iron-scavenging
Elicitors			
Syringolides	+	Acyl glycosides	Elicitor

Table 1 contains a list of toxins, phytohormones, phytohormone mimics, siderophores, and elicitors of unknown virulence function produced by phytopathogenic pseudomonads (based on the literature [3,20,41,46,47,48] and the articles cited therein). ^1^ Phaseolotoxin non-producing isolates were reported in the literature [49]. ^2^ Ethylene-producing strains were isolated from kudzu but not common bean [50]. ^3^ Genes associated with auxin biosynthesis are present in some *Psp* strains [42], and IAA production has been reported in vitro under tryptophan-supplemented conditions [45]; however, alteration of host auxin metabolism or auxin-dependent stomatal opening/reopening during *Psp* infection has not been demonstrated in planta. ^4^ Single coronatine producing isolate has been reported in the literature [40]. ^5^ Yersiniabactin production was not detected by biochemical tests, as reported in the literature [51], although the gene responsible for its production is present. n.d.—no data.

**Table 2:** List of potential, other than *Phaseolus vulgaris* L., hosts and plant reservoirs of *Pseudomonas savastanoi* pv. *phaseolicola* (*Psp*).

Host	Family	Lifestyle of Plant	Lifestyle of *Psp* ^1^	Reference
*Amaranthus retroflexus* L.	Amaranthaceae	A	Epiphytic	[137]
*Chenopodium album* L.	Amaranthaceae	A	Epiphytic	[137]
*Galinsoga parviflora* Cav.	Asteraceae	A	Epiphytic	[137]
*Sonchus oleraceus* L.	Asteraceae	A	Epiphytic	[142]
*Mercurialis annua* L.	Euphorbiaceae	A	Epiphytic	[142]
*Cajanus cajan* (L.) Huth	Fabaceae	A/B	Pathogenic	[10]
*Desmodium* sp.	Fabaceae	Species-dependent	Pathogenic	[10]
*Lablab purpureus* (L.) Sweet	Fabaceae	A	Pathogenic	[10]
*Macroptilium atropurpureum* (DC.) Urb.	Fabaceae	A−P	Pathogenic	[10]
*Neonotonia wightii* (Wight & Arn.) J.A.Lackey	Fabaceae	P	Pathogenic	[10,130]
*Pachyrhizus erosus* (L.) Urb.	Fabaceae	P	Pathogenic	[136]
*Phaseolus acutifolius* A.Gray	Fabaceae	A	Pathogenic	[10]
*Phaseolus coccineus* L.	Fabaceae	A−P	Pathogenic	[10,138]
*Phaseolus lunatus* L.	Fabaceae	A	Pathogenic	[10,138]
*Pueraria lobata* (Willd.) Ohwi	Fabaceae	P	Pathogenic	[50,139]
*Vigna angularis* (Willd.) Ohwi & H.Ohashi	Fabaceae	A	Pathogenic	[10]
*Vigna radiata* (L.) R.Wilczek	Fabaceae	A	Pathogenic	[10,140,141]
*Fumaria* sp.	Papaveraceae	A	Epiphytic	[142]
*Portulaca oleracea* L.	Portulacaceae	A	Epiphytic	[137]
*Datura stramonium* L.	Solanaceae	A	Epiphytic	[137]
*Solanum nigrum* L.	Solanaceae	A	Epiphytic	[137,142]

^1^ For the epiphytic lifestyle category, *Psp* was recovered as epiphytic populations from symptomless plants, with no evidence of pathogenic development on these taxa. Abbreviations: A—annual herb; B—biennial herb; P—perennial herb.

**Table 3:** Stage-specific unresolved questions in the infection strategy of *Pseudomonas savastanoi* pv. *phaseolicola* (*Psp*) and their relevance for future mechanistic studies and halo blight (*HB*) management.

Infection Stage	Question	Reason
Colonization	Does *Psp* actively reopen stomata during bean infection and, if so, is auxin involved?	It is unclear whether stomatal entry is solely opportunistic or can be aided by the pathogen.
Colonization	Do *Psp* cell wall-degrading enzymes contribute considerably to virulence?	It is unclear whether degradation of cell walls supports further infection stages.
Compatible habitat establishment	Is phaseolotoxin-mediated disruption of host ornithine/arginine metabolism an adaptation to destabilize host functioning, satisfy own nutritional demands, or both?	It is not clear whether phaseolotoxin-based toxicity secures arginine supply to pathogens.
Compatible habitat establishment	Can *Psp* use xenosiderophores or other host-/microbiome-derived iron chelators?	It is not clear how triple mutants are able to satisfy their iron demands in planta.
Evasion	Does division of labor in T3SS/effector expression determine the efficiency of immune suppression in planta?	It is not clear how division of labor is regulated to support adequate levels of immunity suppression.
Evasion	Does *Psp* sense host internal volatile cues (e.g., H_2_S) to modulate expression of evasion-associated traits?	It is not clear whether molecular rearrangements in pathogens caused by H_2_S are side effects or an adaptation.
Multiplication	How do epiphytic and internal *Psp* populations interact during infection and spread?	It is not clear whether these two populations compete or cooperate.
Multiplication	Which mechanisms most strongly determine successful multiplication of *Psp* in planta?	It is not clear whether division of labor between subpopulations is the only or major mechanism regulating intra-host spread.
Spread	What is the epidemiological importance of alternative hosts and plant reservoirs?	It is unclear whether alternative hosts and plant reservoirs contribute substantially to disease outbreak.
Spread	Can *Psp* be disseminated by invertebrates?	It is unclear whether invertebrates can contribute to disease outbreaks.

## Data Availability

No new data were created or analyzed in this study. Data sharing is not applicable to this article.

## Abbreviations

The following abbreviations are used in this manuscript:

ABA	abscisic acid
EPS	extracellular polysaccharide
ET	ethylene
flg22	conserved N-terminal part of flagellin
*HB*	halo blight
HR	hypersensitive response
IAA	indole-3-acetic acid
INP	ice nucleation protein
JA	jasmonic acid
LRR	leucine-rich repeat (proteins)
MAMP	microbe-associated molecular pattern
MAPK	mitogen-activated protein kinase
NLR	nucleotide-binding leucine-rich repeat (protein)
ORF	open reading frame
*Psp*	*Pseudomonas savastanoi* pv. *phaseolicola*
PTI	pattern-triggered immunity
QTL	quantitative trait locus
ROS	reactive oxygen species
SA	salicylic acid
T2SS	type two secretion system
T3SS	type three secretion system
T6SS	type six secretion system
